# 1-Tosyl-4-[2-(trifluoro­meth­yl)benz­yl]piperazine

**DOI:** 10.1107/S1600536813000317

**Published:** 2013-01-16

**Authors:** S. Sreenivasa, H. C. Anitha, K. E. ManojKumar, J. Tonannavar, Yenagi Jayashree, P. A. Suchetan, B. S. Palakshamurthy

**Affiliations:** a Department of Studies and Research in Chemistry, Tumkur University, Tumkur, Karnataka 572 103, India; b Department of Physics, Karnatak University, Dharwad, Karnataka 580 003, India; c Department of Studies and Research in Chemistry, UCS, Tumkur University, Tumkur, Karnataka 572 103, India; d Department of Studies and Research in Physics, UCS, Tumkur University, Tumkur, Karnataka 572 103, India

## Abstract

In the crystal structure of the title compound, C_19_H_21_F_3_N_2_O_2_S, the piperazine ring adopts a chair conformation. The dihedral angles between the mean plane of the piperazine ring and the tosyl and trifluoro­methyl­phenyl rings are 74.52 (3) and 68.30 (2)°, respectively. The sulfonamide N atom deviates from the plane defined by the three attached atoms by 0.327 (1) Å. The crystal structure is stabilized by weak C—H⋯π inter­actions.

## Related literature

For the synthesis, characterization and biological activity of piperazine and its derivatives, see: Gan *et al.* (2009*a* ▶,*b* ▶)
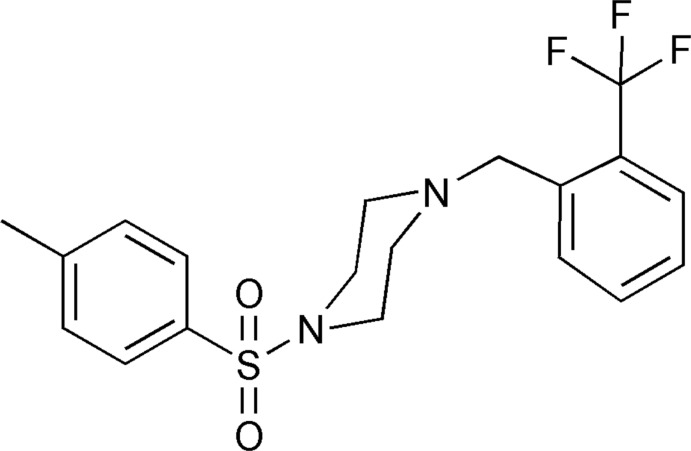


## Experimental

### 

#### Crystal data

C_19_H_21_F_3_N_2_O_2_S
*M* *_r_* = 398.44
Triclinic, 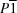

*a* = 9.5044 (3) Å
*b* = 9.8389 (3) Å
*c* = 12.1473 (4) Å
α = 72.036 (1)°
β = 77.024 (1)°
γ = 62.384 (1)°
*V* = 952.96 (5) Å^3^
*Z* = 2
Mo *K*α radiation
μ = 0.22 mm^−1^
*T* = 296 K
0.28 × 0.26 × 0.24 mm

#### Data collection

Bruker APEXII diffractometer
Absorption correction: multi-scan (*SADABS*; Bruker, 2009 ▶) *T*_min_ = 0.942, *T*_max_ = 0.950
18514 measured reflections
3359 independent reflections
2981 reflections with *I* > 2σ(*I*)
*R*_int_ = 0.023

#### Refinement

*R*[*F* ^2^ > 2σ(*F* ^2^)] = 0.042
*wR*(*F* ^2^) = 0.119
*S* = 1.08
3359 reflections
245 parameters
H-atom parameters constrained
Δρ_max_ = 0.18 e Å^−3^
Δρ_min_ = −0.53 e Å^−3^

### 

Data collection: *APEX2* (Bruker, 2009 ▶); cell refinement: *APEX2* and *SAINT-Plus* (Bruker, 2009 ▶); data reduction: *SAINT-Plus* and *XPREP* (Bruker, 2009 ▶); program(s) used to solve structure: *SHELXS97* (Sheldrick, 2008 ▶); program(s) used to refine structure: *SHELXL97* (Sheldrick, 2008 ▶); molecular graphics: *ORTEP-3 for Windows* (Farrugia, 2012 ▶); software used to prepare material for publication: *SHELXL97*.

## Supplementary Material

Crystal structure: contains datablock(s) I, global. DOI: 10.1107/S1600536813000317/gk2548sup1.cif

Structure factors: contains datablock(s) I. DOI: 10.1107/S1600536813000317/gk2548Isup2.hkl

Supplementary material file. DOI: 10.1107/S1600536813000317/gk2548Isup3.cml

Additional supplementary materials:  crystallographic information; 3D view; checkCIF report

## Figures and Tables

**Table 1:** Hydrogen-bond geometry (Å, °) *Cg* is the centroid of the benzene ring of the trifluoro­methyl­phenyl group (C1–C6).

*D*—H⋯*A*	*D*—H	H⋯*A*	*D*⋯*A*	*D*—H⋯*A*
C11—H11*A*⋯*Cg* ^i^	0.97	2.84 (1)	3.670 (2)	144

Symmetry code: (i) 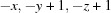.

**Fig. 1.**
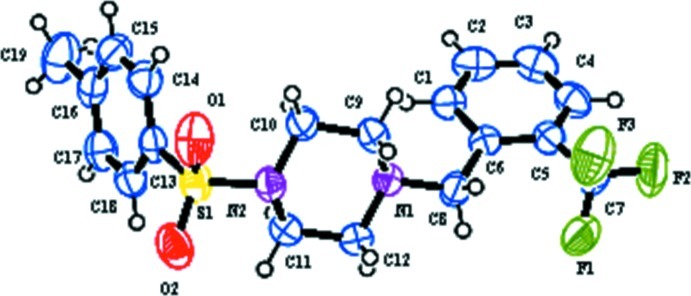
Molecular structure of the title compound. Displacement ellipsoids are drawn at the 50% probability level.

**Fig. 2.**
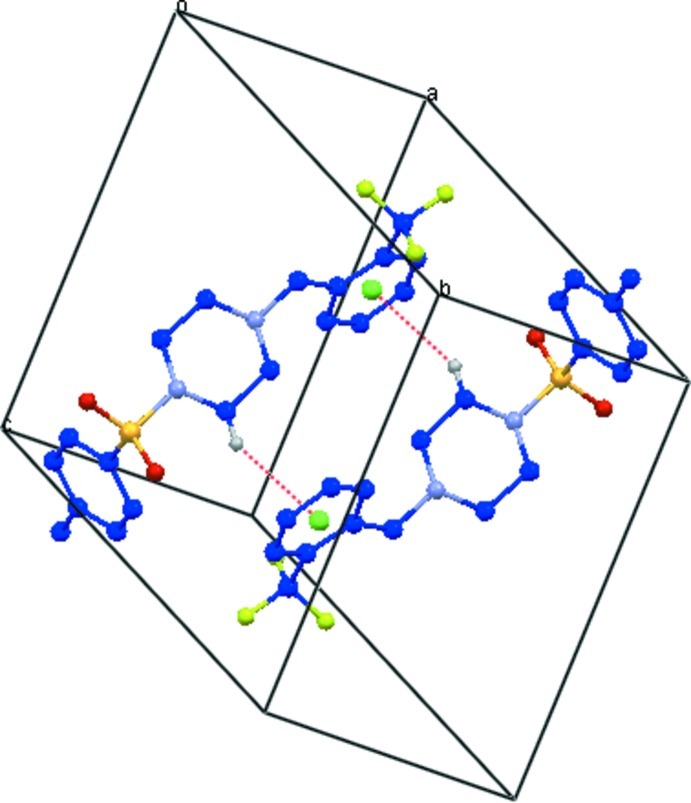
Molecular packing of the title compound. C—H···π interactions are shown as dashed lines.

## supplementary crystallographic information

### Comment

Numerous piperazine derivatives like aryl amide, sulfonamides, Mannich bases, Schiff bases, thiazolidinones, azetidinones, imidazolinones have shown a wide spectrum of biological activities *viz.* anti-inflammatory, antibacterial, antimalarial, anticonvulsant, antipyretic, antitumor, anthelmintics, analgesic, antidepressant, antifungal, antitubercular, anticancer, antidiabetic (Gan *et al.*, 2009*a*,*b*). Keeping this in mind, we synthesized the title compound and here we report its crystal structure.

### Experimental

A mixture of 1-tosylpiperazine (0.01 mmol), potassium carbonate (0.03 mmol) and 2-trifluoromethylbenzyl bromide (0.01 mmol) was added into dry acetonitrile (5 ml). The mixture was stirred at 85°C for 8 h. The reaction was monitored by TLC. Solvent was removed by vacuum distillation and the crude product obtained was purified by column chromatography using 230–400 silica gel and petroleum ether/ethyl acetate as eluent. Single crystals of the title compound were obtained from a mixture of petroleum ether/ethyl acetate (7:3) by slow evaporation technique.

### Refinement

All H atoms were included in calculated positions with C—H bond distances 0.93–0.97 Å and refined in a riding model approximation with *U*_iso_(H) = 1.5*U*_eq_(C_methyl_) and *U*_ĩso_(H) = 1.2*U*_eq_(C) for the remaining H atoms.

### Crystal data

**Table:**

C_19_H_21_F_3_N_2_O_2_S	*F*(000) = 416
*M**_r_* = 398.44	prism
Triclinic, *P*1	*D*_x_ = 1.389 Mg m^−^^3^
Hall symbol: -P 1	Melting point: 455 K
*a* = 9.5044 (3) Å	Mo *K*α radiation, λ = 0.71073 Å
*b* = 9.8389 (3) Å	Cell parameters from 3359 reflections
*c* = 12.1473 (4) Å	θ = 1.8–25.0°
α = 72.036 (1)°	µ = 0.22 mm^−^^1^
β = 77.024 (1)°	*T* = 296 K
γ = 62.384 (1)°	Prism, colourless
*V* = 952.96 (5) Å^3^	0.28 × 0.26 × 0.24 mm
*Z* = 2	

### Data collection

**Table:**

Bruker APEXII diffractometer	2981 reflections with *I* > 2σ(*I*)
Radiation source: fine-focus sealed tube	*R*_int_ = 0.023
Graphite monochromator	θ_max_ = 25.0°, θ_min_ = 1.8°
φ and ω scans	*h* = −11→11
Absorption correction: multi-scan (*SADABS*; Bruker, 2009)	*k* = −11→11
*T*_min_ = 0.942, *T*_max_ = 0.950	*l* = −14→14
18514 measured reflections	2981 standard reflections every 3359 reflections
3359 independent reflections	intensity decay: 0.6%

### Refinement

**Table:**

Refinement on *F*^2^	Primary atom site location: structure-invariant direct methods
Least-squares matrix: full	Secondary atom site location: difference Fourier map
*R*[*F*^2^ > 2σ(*F*^2^)] = 0.042	Hydrogen site location: inferred from neighbouring sites
*wR*(*F*^2^) = 0.119	H-atom parameters constrained
*S* = 1.08	*w* = 1/[σ^2^(*F*_o_^2^) + (0.0669*P*)^2^ + 0.2061*P*] where *P* = (*F*_o_^2^ + 2*F*_c_^2^)/3
3359 reflections	(Δ/σ)_max_ = 0.001
245 parameters	Δρ_max_ = 0.18 e Å^−^^3^
0 restraints	Δρ_min_ = −0.53 e Å^−^^3^
0 constraints	

### Special details

**Table:**

Geometry. All e.s.d.'s (except the e.s.d. in the dihedral angle between two l.s. planes) are estimated using the full covariance matrix. The cell e.s.d.'s are taken into account individually in the estimation of e.s.d.'s in distances, angles and torsion angles; correlations between e.s.d.'s in cell parameters are only used when they are defined by crystal symmetry. An approximate (isotropic) treatment of cell e.s.d.'s is used for estimating e.s.d.'s involving l.s. planes.
Refinement. Refinement of *F*^2^ against ALL reflections. The weighted *R*-factor *wR* and goodness of fit *S* are based on *F*^2^, conventional *R*-factors *R* are based on *F*, with *F* set to zero for negative *F*^2^. The threshold expression of *F*^2^ > σ(*F*^2^) is used only for calculating *R*-factors(gt) *etc*. and is not relevant to the choice of reflections for refinement. *R*-factors based on *F*^2^ are statistically about twice as large as those based on *F*, and *R*-factors based on ALL data will be even larger.

### Fractional atomic coordinates and isotropic or equivalent isotropic displacement parameters (Å^2^)

**Table:**

	*x*	*y*	*z*	*U*_iso_*/*U*_eq_	
C1	−0.1973 (2)	0.79085 (19)	1.04506 (16)	0.0524 (4)	
H1	−0.1413	0.8051	0.9723	0.063*	
C2	−0.3565 (2)	0.8200 (2)	1.0527 (2)	0.0650 (5)	
H2	−0.4054	0.8512	0.9852	0.078*	
C3	−0.4417 (2)	0.8030 (2)	1.1589 (2)	0.0692 (6)	
H3	−0.5494	0.8269	1.1637	0.083*	
C4	−0.3681 (2)	0.7507 (2)	1.25786 (19)	0.0601 (5)	
H4	−0.4258	0.7382	1.3301	0.072*	
C5	−0.2083 (2)	0.71618 (19)	1.25148 (15)	0.0484 (4)	
C6	−0.12093 (19)	0.74094 (18)	1.14417 (14)	0.0446 (4)	
C7	−0.1307 (2)	0.6477 (3)	1.36242 (17)	0.0662 (5)	
C8	0.0483 (2)	0.7240 (3)	1.13458 (15)	0.0570 (4)	
H8A	0.0465	0.8048	1.1654	0.068*	
H8B	0.1120	0.6221	1.1819	0.068*	
C9	0.1988 (2)	0.5868 (2)	0.98362 (15)	0.0533 (4)	
H9A	0.1208	0.5447	0.9957	0.064*	
H9B	0.2835	0.5124	1.0328	0.064*	
C10	0.2667 (2)	0.6058 (2)	0.85784 (15)	0.0512 (4)	
H10A	0.3190	0.5041	0.8378	0.061*	
H10B	0.1818	0.6756	0.8079	0.061*	
C11	0.3078 (2)	0.8246 (2)	0.87272 (15)	0.0549 (4)	
H11A	0.2231	0.8998	0.8237	0.066*	
H11B	0.3862	0.8658	0.8617	0.066*	
C12	0.2407 (2)	0.8003 (2)	0.99864 (16)	0.0572 (4)	
H12A	0.3265	0.7275	1.0473	0.069*	
H12B	0.1908	0.9000	1.0213	0.069*	
C13	0.39911 (19)	0.7588 (2)	0.60451 (14)	0.0494 (4)	
C14	0.3464 (2)	0.6848 (2)	0.55409 (16)	0.0586 (5)	
H14	0.3724	0.5776	0.5826	0.070*	
C15	0.2560 (2)	0.7693 (3)	0.46208 (17)	0.0650 (5)	
H15	0.2211	0.7187	0.4284	0.078*	
C16	0.2156 (2)	0.9293 (3)	0.41834 (15)	0.0607 (5)	
C17	0.2694 (2)	1.0018 (2)	0.46948 (16)	0.0623 (5)	
H17	0.2440	1.1088	0.4407	0.075*	
C18	0.3601 (2)	0.9183 (2)	0.56233 (16)	0.0564 (4)	
H18	0.3947	0.9687	0.5964	0.068*	
C19	0.1146 (3)	1.0231 (4)	0.3176 (2)	0.0932 (8)	
H19A	0.0038	1.0616	0.3464	0.140*	
H19B	0.1364	0.9567	0.2665	0.140*	
H19C	0.1394	1.1108	0.2758	0.140*	
N1	0.12372 (17)	0.73734 (17)	1.01560 (12)	0.0492 (3)	
N2	0.38211 (16)	0.67220 (16)	0.84130 (12)	0.0482 (3)	
O1	0.58136 (16)	0.48817 (17)	0.72513 (13)	0.0758 (4)	
O2	0.60748 (16)	0.7253 (2)	0.72858 (12)	0.0771 (4)	
F1	−0.05597 (17)	0.7266 (2)	1.37482 (11)	0.0944 (5)	
F2	−0.23300 (18)	0.6445 (2)	1.45635 (11)	0.1050 (5)	
F3	−0.02138 (19)	0.49998 (18)	1.36833 (12)	0.1020 (5)	
S1	0.51074 (5)	0.65163 (6)	0.72555 (4)	0.05661 (18)	

### Atomic displacement parameters (Å^2^)

**Table:**

	*U*^11^	*U*^22^	*U*^33^	*U*^12^	*U*^13^	*U*^23^
C1	0.0562 (10)	0.0455 (9)	0.0529 (10)	−0.0191 (8)	−0.0149 (8)	−0.0053 (7)
C2	0.0602 (11)	0.0482 (10)	0.0846 (14)	−0.0144 (9)	−0.0344 (11)	−0.0076 (9)
C3	0.0427 (10)	0.0585 (11)	0.1037 (17)	−0.0164 (9)	−0.0127 (11)	−0.0198 (11)
C4	0.0473 (10)	0.0564 (10)	0.0756 (13)	−0.0231 (8)	0.0041 (9)	−0.0199 (9)
C5	0.0462 (9)	0.0456 (9)	0.0517 (9)	−0.0183 (7)	−0.0002 (7)	−0.0144 (7)
C6	0.0433 (8)	0.0414 (8)	0.0475 (9)	−0.0150 (7)	−0.0060 (7)	−0.0121 (7)
C7	0.0612 (12)	0.0861 (15)	0.0477 (10)	−0.0313 (11)	0.0032 (9)	−0.0172 (10)
C8	0.0502 (10)	0.0831 (13)	0.0427 (9)	−0.0317 (9)	−0.0003 (7)	−0.0193 (9)
C9	0.0600 (10)	0.0541 (10)	0.0493 (9)	−0.0324 (9)	0.0011 (8)	−0.0084 (7)
C10	0.0595 (10)	0.0490 (9)	0.0479 (9)	−0.0269 (8)	0.0021 (8)	−0.0139 (7)
C11	0.0650 (11)	0.0588 (10)	0.0516 (10)	−0.0377 (9)	−0.0018 (8)	−0.0115 (8)
C12	0.0670 (11)	0.0690 (11)	0.0508 (10)	−0.0406 (10)	0.0005 (8)	−0.0199 (8)
C13	0.0414 (8)	0.0590 (10)	0.0397 (8)	−0.0213 (8)	0.0039 (7)	−0.0068 (7)
C14	0.0609 (11)	0.0564 (10)	0.0553 (10)	−0.0245 (9)	0.0012 (8)	−0.0146 (8)
C15	0.0638 (12)	0.0822 (14)	0.0566 (11)	−0.0333 (11)	−0.0024 (9)	−0.0254 (10)
C16	0.0500 (10)	0.0817 (14)	0.0409 (9)	−0.0236 (9)	−0.0002 (7)	−0.0119 (9)
C17	0.0643 (12)	0.0602 (11)	0.0505 (10)	−0.0255 (9)	−0.0038 (9)	−0.0002 (8)
C18	0.0604 (11)	0.0619 (11)	0.0494 (10)	−0.0326 (9)	−0.0038 (8)	−0.0068 (8)
C19	0.0775 (15)	0.124 (2)	0.0571 (13)	−0.0275 (15)	−0.0203 (11)	−0.0076 (13)
N1	0.0514 (8)	0.0605 (8)	0.0421 (7)	−0.0291 (7)	0.0010 (6)	−0.0160 (6)
N2	0.0475 (8)	0.0526 (8)	0.0414 (7)	−0.0232 (6)	−0.0020 (6)	−0.0054 (6)
O1	0.0583 (8)	0.0634 (8)	0.0647 (9)	−0.0005 (7)	0.0021 (6)	−0.0076 (6)
O2	0.0539 (8)	0.1138 (12)	0.0621 (8)	−0.0461 (8)	−0.0073 (6)	−0.0008 (8)
F1	0.0980 (10)	0.1560 (14)	0.0594 (8)	−0.0745 (10)	−0.0026 (7)	−0.0361 (8)
F2	0.0923 (10)	0.1675 (15)	0.0502 (7)	−0.0651 (10)	0.0168 (7)	−0.0202 (8)
F3	0.1030 (11)	0.0919 (10)	0.0645 (8)	−0.0078 (8)	−0.0260 (7)	0.0014 (7)
S1	0.0409 (3)	0.0670 (3)	0.0460 (3)	−0.0177 (2)	−0.00155 (18)	−0.0027 (2)

### Geometric parameters (Å, º)

**Table:**

C1—C6	1.384 (2)	C11—N2	1.464 (2)
C1—C2	1.390 (3)	C11—C12	1.510 (2)
C1—H1	0.9300	C11—H11A	0.9700
C2—C3	1.368 (3)	C11—H11B	0.9700
C2—H2	0.9300	C12—N1	1.456 (2)
C3—C4	1.364 (3)	C12—H12A	0.9700
C3—H3	0.9300	C12—H12B	0.9700
C4—C5	1.384 (2)	C13—C14	1.381 (3)
C4—H4	0.9300	C13—C18	1.382 (3)
C5—C6	1.398 (2)	C13—S1	1.7625 (17)
C5—C7	1.496 (3)	C14—C15	1.369 (3)
C6—C8	1.519 (2)	C14—H14	0.9300
C7—F2	1.325 (2)	C15—C16	1.386 (3)
C7—F1	1.328 (3)	C15—H15	0.9300
C7—F3	1.329 (3)	C16—C17	1.383 (3)
C8—N1	1.458 (2)	C16—C19	1.510 (3)
C8—H8A	0.9700	C17—C18	1.378 (3)
C8—H8B	0.9700	C17—H17	0.9300
C9—N1	1.451 (2)	C18—H18	0.9300
C9—C10	1.510 (2)	C19—H19A	0.9600
C9—H9A	0.9700	C19—H19B	0.9600
C9—H9B	0.9700	C19—H19C	0.9600
C10—N2	1.468 (2)	N2—S1	1.6391 (14)
C10—H10A	0.9700	O2—S1	1.4223 (15)
C10—H10B	0.9700	S1—O1	1.4282 (15)
			
C6—C1—C2	120.91 (18)	N2—C11—H11B	110.0
C6—C1—H1	119.5	C12—C11—H11B	110.0
C2—C1—H1	119.5	H11A—C11—H11B	108.4
C3—C2—C1	120.35 (18)	N1—C12—C11	110.19 (14)
C3—C2—H2	119.8	N1—C12—H12A	109.6
C1—C2—H2	119.8	C11—C12—H12A	109.6
C4—C3—C2	119.77 (18)	N1—C12—H12B	109.6
C4—C3—H3	120.1	C11—C12—H12B	109.6
C2—C3—H3	120.1	H12A—C12—H12B	108.1
C3—C4—C5	120.43 (18)	C14—C13—C18	120.00 (17)
C3—C4—H4	119.8	C14—C13—S1	120.06 (14)
C5—C4—H4	119.8	C18—C13—S1	119.91 (14)
C4—C5—C6	120.86 (17)	C15—C14—C13	119.93 (18)
C4—C5—C7	118.11 (16)	C15—C14—H14	120.0
C6—C5—C7	121.01 (15)	C13—C14—H14	120.0
C1—C6—C5	117.53 (15)	C14—C15—C16	121.02 (18)
C1—C6—C8	120.23 (15)	C14—C15—H15	119.5
C5—C6—C8	122.15 (15)	C16—C15—H15	119.5
F2—C7—F1	105.93 (17)	C17—C16—C15	118.43 (18)
F2—C7—F3	106.64 (18)	C17—C16—C19	120.4 (2)
F1—C7—F3	106.07 (18)	C15—C16—C19	121.1 (2)
F2—C7—C5	113.14 (17)	C18—C17—C16	121.17 (18)
F1—C7—C5	113.06 (17)	C18—C17—H17	119.4
F3—C7—C5	111.48 (16)	C16—C17—H17	119.4
N1—C8—C6	113.17 (14)	C17—C18—C13	119.45 (17)
N1—C8—H8A	108.9	C17—C18—H18	120.3
C6—C8—H8A	108.9	C13—C18—H18	120.3
N1—C8—H8B	108.9	C16—C19—H19A	109.5
C6—C8—H8B	108.9	C16—C19—H19B	109.5
H8A—C8—H8B	107.8	H19A—C19—H19B	109.5
N1—C9—C10	110.58 (13)	C16—C19—H19C	109.5
N1—C9—H9A	109.5	H19A—C19—H19C	109.5
C10—C9—H9A	109.5	H19B—C19—H19C	109.5
N1—C9—H9B	109.5	C9—N1—C12	109.73 (14)
C10—C9—H9B	109.5	C9—N1—C8	111.63 (14)
H9A—C9—H9B	108.1	C12—N1—C8	111.32 (13)
N2—C10—C9	108.49 (13)	C11—N2—C10	111.46 (13)
N2—C10—H10A	110.0	C11—N2—S1	118.22 (11)
C9—C10—H10A	110.0	C10—N2—S1	116.83 (11)
N2—C10—H10B	110.0	O2—S1—O1	120.28 (9)
C9—C10—H10B	110.0	O2—S1—N2	106.27 (8)
H10A—C10—H10B	108.4	O1—S1—N2	106.46 (8)
N2—C11—C12	108.27 (14)	O2—S1—C13	108.64 (8)
N2—C11—H11A	110.0	O1—S1—C13	107.92 (9)
C12—C11—H11A	110.0	N2—S1—C13	106.48 (7)

### Hydrogen-bond geometry (Å, º)

Cg is the centroid of the benzene ring of the trifluoromethylphenyl group (C1–C6).

**Table:**

*D*—H···*A*	*D*—H	H···*A*	*D*···*A*	*D*—H···*A*
C11—H11*A*···*Cg*^i^	0.97	2.84 (1)	3.670 (2)	144

Symmetry code: (i) −*x*, −*y*+1, −*z*+1.
